# Current status of indocyanine green fluorescent angiography in assessing perfusion of gastric conduit and oesophago-gastric anastomosis

**DOI:** 10.1097/JS9.0000000000000913

**Published:** 2023-11-20

**Authors:** Syed Nusrath, Prasanthi Kalluru, Srijan Shukla, Anvesh Dharanikota, Madhunarayana Basude, Pawan Jonnada, Muayyad Abualjadayel, Saleh Alabbad, Tanveer Ahmad Mir, Dieter C. Broering, KVVN Raju, Thammineedi Subramanyeshwar Rao, Yogesh Kumar Vashist

**Affiliations:** Departrments of a Surgical Oncology; b Clinical Research, Basavatarakam Indo American Cancer Hospital and Research Institute, Hyderabad, India; c Tissue/Organ Bioengineering & BioMEMS Laboratory, TR&I Department; d Organ Transplant Center of Excellence, King Faisal Specialist Hospital and Research Center, Riyadh, Kingdom of Saudi Arabia

**Keywords:** anastomotic leak, oesophageal cancer surgery, gastric conduit, Indocyanine green fluorescent angiography, morbidity, perfusion

## Abstract

Anastomotic leak (AL) remains a significant complication after esophagectomy. Indocyanine green fluorescent angiography (ICG-FA) is a promising and safe technique for assessing gastric conduit (GC) perfusion intraoperatively. It provides detailed visualization of tissue perfusion and has demonstrated usefulness in oesophageal surgery. GC perfusion analysis by ICG-FA is crucial in constructing the conduit and selecting the anastomotic site and enables surgeons to make necessary adjustments during surgery to potentially reduce ALs. However, anastomotic integrity involves multiple factors, and ICG-FA must be combined with optimization of patient and procedural factors to decrease AL rates. This review summarizes ICG-FA’s current applications in assessing esophago-gastric anastomosis perfusion, including qualitative and quantitative analysis and different imaging systems. It also explores how fluorescent imaging could decrease ALs and aid clinicians in utilizing ICG-FA to improve esophagectomy outcomes.

Highlights

- Anastomotic leakage is frequent in oesophageal cancer surgery.
- Gastric conduit perfusion is dependent mainly on the right gastroepiploic artery.
- About half to one-third of the gastric conduit has an intramural blood supply.
- Indocyanine green fluorescent angiography is a useful tool for assessing gastric conduit perfusion.
- Indocyanine green fluorescent angiography helps for choice of anastomotic site.
- Indocyanine green fluorescent angiography can potentially reduce anastomotic leak.

## Introduction

Surgical resection is the primary treatment approach for early and locally advanced oesophageal cancer^1^. Despite advancements in minimally invasive surgery (MIS) and improved postoperative care, esophagectomy continues to exhibit a notable elevated morbidity rate^2^. Among its complications, anastomotic leak (AL) is one of the most significant occurring following Ivor Lewis and McKeown esophagectomy. Its incidence remains substantial, reaching upto 40%—depending on the site of the anastomosis—even in high-volume centres^3–6^.

AL is not only linked to increased postoperative mortality but also results in prolonged hospital stay and a diminished quality of life (QoL) for patients^7^. In an analysis of the Society of Thoracic Surgeons (STS) general thoracic surgery database, the 30-day mortality rate for patients undergoing esophagectomy was reported with 3.3% and besides respiratory distress syndrome, reintubation and renal failure, AL was identified as one of the most significant trigger for operative mortality. Notably, the overall AL rate in this study was 12.8%^8^. However, in another separate STS database study that examined AL rates based on the site of the anastomosis, it was found that a cervical anastomosis had a 12.3% anastomotic leak rate, while a thoracic anastomosis had a slightly lower rate of 9.3% only. Despite these variations in AL rates, both groups exhibited similar 30-day mortality rates of 3.6% and 2.7%, respectively^9^. From the clinical management point of view the resulting condition of a mediastinal AL compared to a AL in the neck area is different, hence many high-volume centres prefer to perform an anastomosis in the neck rather than mediastinum. ALs are further associated with complications such as arrhythmias, deep venous thrombosis, pneumonia, acute respiratory distress syndrome, the need for ventilatory support, empyema, sepsis, stricture formation, and renal failure. Furthermore, AL has been associated with local recurrence and inferior long-term oncological outcomes^10,11^.

While several risk factors for AL have been discussed and those may differ between mediastinal and neck anastomoses, one of the most significant factors appears to be an insufficient perfusion of the gastric conduit (GC). During esophagectomy, GC is the most common used substitute for the esophageus. The blood supply to the GC relies entirely on the right gastroepiploic artery (RGEA), and the distal-most portion of the GC experiences relatively reduced blood flow since the RGEA immerses into the gastric wall and the entire proximal region of the GC has only an intramural perfusion. Submucosal vessels are the primary source of blood supply for this section of the GC. Therefore, placing the anastomosis in well-perfused area of the GC appears to be critical to reduce AL rates.

An emerging method, near-infrared Indocyanine green fluorescence angiography (ICG-FA), helps in identifying well-perfused region of GC for basing anastomosis and shows promise with good intraoperative decision-making^12,13^.

This review summarizes the current applications of ICG-FA in the perfusion assessment of esophago-gastric anastomosis (EGA) after esophagectomy and reconstruction using a GC. We attempted to include both qualitative and quantitative analyses and reviews as well as available imaging systems to cover the entire spectrum for successful ICG-FA usage in clinical routine.

## Methods

**Figure 1:**
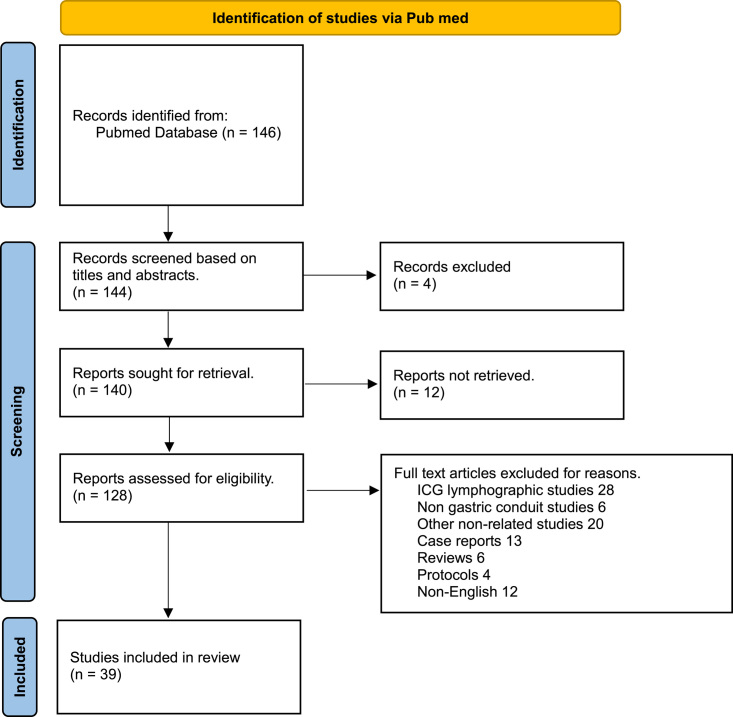
The PRISMA flowchart summarizes the comprehensive research and selection process. ICG, indocyanine green.

In July 2023 we conducted a comprehensive systematic review utilizing the PubMed database—the search was conducted in conformity with the Preferred Reporting Items for Systematic Reviews and Meta-Analyses (PRISMA) guidelines in general, but we opt not to exclude any full text report with original data available in English language. The use of ICG fluorescence in patients was identified through PubMed database search, employing the medical-subject-headings terms “esophagectomy” AND “Indocyanine green” OR “fluorescence guided” OR “gastric tube” OR “gastric conduit”. Studies were included in the analysis if full text manuscripts were published in English language and only original data presented. Initial screening for eligibility based on title and abstract was conducted by two authors (S.N. and Y.K.V.). When potentially relevant articles were found, both authors independently assessed the full texts to determine which ones to include in the final review. Furthermore, additional records were discovered by manually reviewing the references cited in each selected article. In cases where there were disagreements in study selection, consensus was reached through a re-review process involving the two reviewers. Figure 1 summarizes our selection process in form of a PRSIMA flowchart.

## Results

### Encounter the clinical challenge—a glimpse of light—green light

Historically, evaluation of GC perfusion relied on the surgeon’s visual and manual assessments, including factors such as colour, bleeding from cut edges, peristalsis, and the pulse palpation of the RGEA. Several studies have highlighted the limited accuracy of clinical criteria based on intestinal colour, arterial pulsation, and peristalsis for predicting AL in colorectal surgeries. Additionally, these criteria are also less reliable for assessing intestinal viability during acute intestinal ischaemia and estimating GC perfusion in esophagectomy patients^14–16^.

Several strategies and techniques have been developed to assess and enhance blood flow in the GC, aiming to minimize postoperative complications. One such strategy involves gastric ischaemic preconditioning before oesophageal surgery. This approach seeks to redistribute gastric blood supply preoperatively, leading to improved tissue oxygenation at the distal conduit for a better EGA site, ultimately reducing postoperative AL rates. During ischaemic preconditioning, a portion of the stomach’s blood supply is intentionally restricted by embolization of either the left gastric artery or short gastric vessels or both before surgery. This restriction encourages the formation of collateral vessels, which in turn augment blood flow to the distal regions of the GC^17–19^.

Although ischaemic preconditioning may seem like an appealing strategy, multiple studies, including a randomized trial, have failed to prove its effectiveness in reducing the rate of ALs^4,6^. Despite various attempts to predict the risk of anastomotic complications by assessing GC perfusion during surgery using a range of methods, none have yet been successfully translated in clinical practice. These methods include intramucosal pH, pulse-oxymetry, mucosal CO2, Doppler spectroscopy, near-infrared and visible light spectro-photometry, infrared imaging, laser Doppler flowmetry, and bowel wall contractility measurements^20–24^.

However, a more recent and promising technique is the fluorescent imaging, specifically the use of ICG. Fluorescence imaging involves the injection of a small amount of fluorescent dye into the patient at a precise point in the procedure. Typically, this procedure employs a specialized camera equipped with a dedicated fluorescent light source and sensor. The fluorescent light source emits light at a specific wavelength designed to activate the fluorescent dye, causing it to emit light at a known wavelength, which is then captured by the fluorescent sensor. This fluorescence image can be observed either on its own or overlaid onto a standard laparoscopic/ thoracoscopic image, offering real-time visualization of organ perfusion. This technique provides a wider view of tissue perfusion and has shown to be valuable in both oesophageal and colorectal surgery for a better intraoperative decision-making^25–29^.

**Figure 2:**
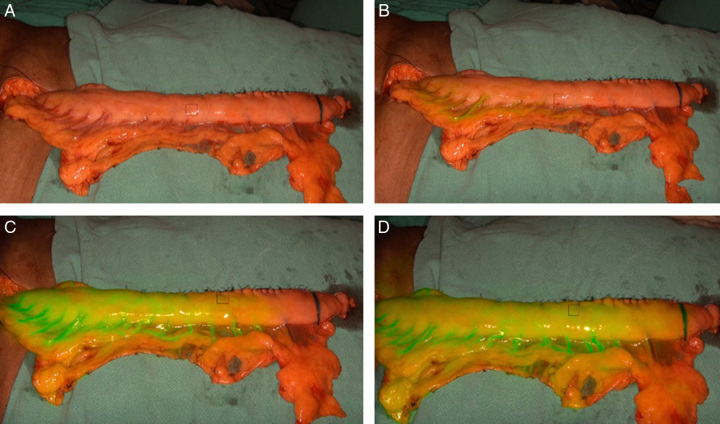
Indocyanine green (ICG) fluorescence angiography of gastric conduit. (A) Perfusion of gastric conduit in white light. Note the part of conduit distal to inked line is dusky with doubtful vascularity by visual inspection. (B). At 7 sec after IV injection in central venous catheter, the first appearance of ICG blush in gastric conduit is seen along the gastroepiploic arterial arcade. (C) At 15 sec after injection almost 2/3^rd^ of the gastric conduit demonstrate ICG colour. (D) At 25 sec post-injection still the distal few cms of the gastric conduit is not enhancing with ICG fluorescence but the space between the black marking and the last arcade before entering the gastric conduit wall, now demonstrates the submucosal perfusion.

By injecting ICG into the bloodstream, it fluoresces under near-infrared light, enabling surgeons to view tissue blood flow in real-time and make necessary adjustments to potentially reduce ALs by highlighting ischaemic area. Figure 2A-D demonstrate the intraoperative real-time ICG-FA of a GC at several time points and clearly differentiates between the well-perfused area through the RGEA, the submucosal perfused and non-perfused zone. The efficacy of ICG-FA in predicting free flap necrosis by monitoring the perfusion index was demonstrated in rat models by Giunta and colleagues, and the findings were validated in humans by Lamby and Prantl^30–32^. Additionally, Kudszus and colleagues were the first to describe the usefulness of ICG-FA in assessing perfusion in colorectal surgery, while Murawa and colleagues first reported its value in evaluating perfusion in EGA^13,33^. The safety, wider availability, and ease of use of fluorescent imaging using ICG have made it increasingly popular. ICG-FA can aid in identifying well-perfused region of GC for basing EGA. This especially applies when the anastomosis is performed in the neck region due to the length of the GC.

ICG is a hydrophilic, tri-carbocyanine fluorescent dye that has become increasingly popular in various surgical fields due to its ability to produce high-quality images with good tissue contrast and sensitivity. ICG has a low inherent auto-fluorescence background, which makes it an excellent choice for imaging. Its excitation spectrum ranges from 700 to 850 nm, while its emission spectrum peaks between 810 and 830 nm, which enables easy detection and distinction from other tissues. The lyophilized powder form of ICG is easily reconstituted in distilled water and can be diluted with a saline solution before injection. Its ability to penetrate deep into tissues makes it a popular choice for a variety of surgical applications^34^.

In fluorescence angiography, ICG is typically injected as an intravenous bolus through a peripheral intravenous line or a central venous line, followed by a flush to ensure that the dye is pushed into circulation as a bolus. The compound binds rapidly to plasma proteins in the blood and is quickly cleared by the liver and excreted in the biliary system. Its half-life is two to four minutes, and it is usually cleared from the blood in 15 to 20 min^12,27,35^. Detection of the dye usually occurs within seconds after injection, although the time to fluorescence after injection may be affected by factors such as infusion type, injection site, ICG dose, haematocrit, and cardiac output^27,35^.

ICG was approved by the FDA for human use since 1956 and has since than been a valuable tool in both clinical and research settings. Initially, its applications were limited, gaining approval for neurosurgical research in 2003 and cardiac vessel angiography in 2005. Approval for use with surgical microscopes followed in 2006. ICG has also received approval for ophthalmic angiography and has been widely used off-label for real-time imaging across various fields, including abdominal and plastic surgery, oncological staging, and perfusion assessment. Devices employing ICG-FA have obtained FDA clearance based on their similarity to previously approved ICG applications, without the need for additional documentation of clinical effectiveness^36^.

### Application, measurement, and stratification

**Figure 3:**
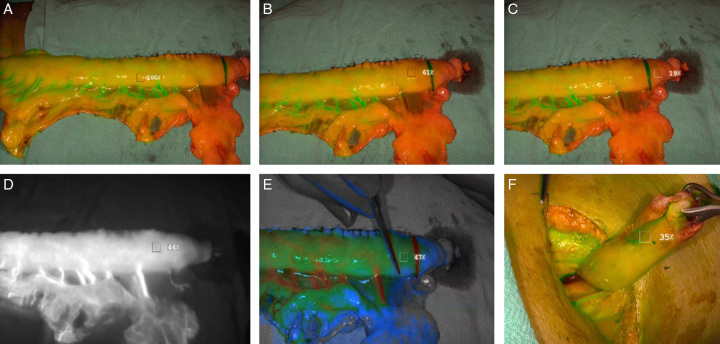
Quantification perfusion analysis. (A) A reference point on well-vascularized part of gastric conduit is selected. Here 10 cms from pylorus on conduit is marked as 100% (Seen in overlay mode). (B) Relative perfusion of 61% (in relation to reference point) 2 cm proximal to inked line (visual boundary of poor perfusion). Seen in overlay mode. (C) Perfusion distal to inked line with 19% relative perfusion. Seen in overlay mode. (D) Borderline perfusion of conduit (46%) just proximal to inked line as seen in fluorescent mode. (E) Borderline perfusion of conduit (47%) just proximal to inked line in colour-segmented fluorescent (CSF) mode. Grey colour indicates avascular zone. Blue poorly perfused zone. Green and Red or pink indicate well perfused. (F) Perfusion of oesophageal stump at the tip (35%) in overlay mode.

Application, measurement, stratification, and outcome with ICG-FA imaging systems that enable real-time fluorescence imaging are available for both minimally invasive and open surgical procedures. Certain imaging systems are equipped with software that can measure the fluorescent intensity (FI) at specific regions of interest. Table 1 provides a summary of the imaging systems and software used for ICG-FA for EGA. Figure 3A-F exemplarily reveals GC in different modes and also depicts measurement of perfusion quantification by ICF-FA using Strykers Spy-phi device.

**Table 1:** Commonly used devices for ICG-FA imaging.

Manufacturer	Imaging system	Light source	Utility	Software	Modes available
Mizuho Ikakogyo Co., Ltd, Tokyo, Japan	HyperEye Medical System	LED	Portable hand held imaging device for OP	LumiView	1.Superimposed fluorescence image on colour images
Hamamatsu Photonics K.K, Hamamatsu, Japan	Photodynamic eye‐Neo II	LED	Portable hand held imaging device for OP	ROIs	1. Colour and black and white fluorescent image 2. Fluorescence mapping function 3. Focus adjustment (near ‐ far)
Novadaq Technologies, Ontario, Canada	SPY Elite Imaging System (Integrated with Stryker’s)	Laser	Laparoscopic, Thoracoscopic surgery	SPY-Q	
Da Vinci Surgical Systems Intuitive Surgical, Sunnyvale, CA	Firefly camera systems integrated into da Vinci Si and Xi surgical robots (Intuitive Surgical Inc)	3D LED illuminator	Robotic surgery	Da Vinci OS4	1. Firefly Si (camera at the end of a laparoscope) and Xi (chip ‐ on—a—tip arrangement) 2. Normal imaging and fluorescent modes 3. Firefly is an add ‐ on feature with the Si robot and a standard feature with the Xi robot
Karl Storz, Tuttlingen, Germany	IMAGE1 S Rubina (Integrated 4 K and 3D)	Laser-free LED (Xenon light source)	Laparoscopic, Thoracoscopic surgery	ROIs	1. Overlay mode—blue or green- regular white light image is combined with the NIR/ICG data 2. Intensity map- Displays the intensity of the NIR/ICG signal using a colour scale in an overlay image. 3. Monochromatic- NIR/ICG signal alone is displayed in white on a black background
Karl Storz, Tuttlingen, Germany	Image 1 S system	HD xenon light source (D-light P system)	Laparoscopic, Thoracoscopic surgery	ROIs	1. Optical illumination and contrast enhancement with IMAGE1 S ™ CLARA and CHROMA 2. A foot switch between white light and ICG modes 3. No overlay mode
Stryker, Kalamazoo, MI, USA	1688 AIM (Advanced imaging modality)	LED (L11 light source and auto-light technology)	Laparoscopic, Thoracoscopic surgery	SPY-Q	1. Green overlay mode (superimposed fluorescence on visible light field) 2. SPY ‐ ENV mode (grayscale and green imaging) 3. SPY ‐ Contrast (High contrast visualization of 4K fluorescence in black and white) 4. IRIS (for lighted ureteric stents)
Stryker, Kalamazoo, MI, USA	SPY ‐ PHI	L11 with Auto-Light	Portable hand held imaging device for OP	SPY-Q	1. Green overlay mode 2. Colour-segmented fluorescence mode (gradient proportionate to the intensity of fluorescence. Orange (maximum); Grey (minimum) 3. SPY ‐ Fluorescence mode (white fluorescence against a black background)
Beth Israel Deaconess Medical Center, Boston, MA, USA	Mini-FLARE™	LED	Portable cart based imaging system for OP	Mini-FLARE^TM^ Imaging System Software	Colour Video, NIR Florescence image, Colour- NIR Merge
Fluoptics, Grenoble FRANCE	Fluobeam	Laser	Portable hand held imaging device for OP	.FLUSOFT imaging software	1. Florescence is depicted in black and white images. 2. FLUSOFT imaging software permits pseudo-colouring of fluorescence intensities
Quest medical imaging, Middenmeer, the Netherlands	Quest Spectrum Platform®	LED	Open and laparoscopic procedures	Quest Research Framework®	1. Overlay mode 2. Intensity map

HD, high definition; ICG-FA, indocyanine green fluorescent angiography; LED, light emitting diode; OP, open procedures; ROI, region of interest.

Besides the imaging system also the dose of ICG applied is of importance, however there is no defined dose based on comparative clinical trials. The optimal ICG dose for evaluating the GC varies in different studies, with ranges spanning from 1.25 to 25 mg per bolus (see Table 2). Due to the short half-life of ICG, administration of repeated doses during same surgery is practical. Generally, a bolus injection is given, and additional doses are contemplated when the signal strength is insufficient or unclear. To ensure accuracy in measurements, it is advisable to employ the lowest effective ICG dose, as high doses may lead to background signal interference, which could compromise the precision of the readings^57^.

**Table 2:** ICG-FA assessment of perfusion of GC.

Author (Ref.)	Year	Study design	Country	N	AL in ICG-arm (%)	AL in non-ICG-arm (%)	Operative technique	Timing of ICG Injection	Dose(mg)	Imaging system	AS
Campbell^37^	2015	R	USA	90	0/30 (0)a	12/60(20)	MI-ILE	After GC	5	SPY Elite	T
Dalton^38^	2017	R	USA	40	2/20 (10)	0/20	MI-ILE	After GC	7.5	PINPOINT	T
DeGroot^39^	2022	P	NL	63	14/63(22.2)	—	RA-ILE	After GC	7.5	Firefly	T
Hodari^40^	2015	R	USA	54	0/39 (0)	3/15 (20)	RA-ILE	After GC	NS	Firefly	T
Galema^41^	2023	P	NL	20	3/20 (15)	—	NS	After GC	5	Quest	N
Ishikawa^42^	2021	R	USA	304	70/304 (23)	—	ILE/THE	After GC	5	SPY Elite	N
Karampinis^43^	2017	R	Germany	90	1/35(3)	10/55(20)	MKE/IVE	After GC	7.5	PINPOINT	T/N
Kitagawa^44^	2017	R	Japan	72	7/72 (9.7)	—	MIE	Before & After GC	5	HEMS	N
Koyanagi^45^	2016	R	Japan	40	7/40 (17.5)	—	Open	After GC	1.25/2.5	PDE	N
Kumagai^46^	2018	R	Japan	70	1/70 (1.43)	—	TTE	After GC	2.5	PDE	N
Luo^47^	2021	R	China	192	1/86 (1.16)	11/106 (10.3)	M K MIE	After GC	0.5 mg/kg	Novadaq	N
Murawa^13^	2012	R	Poland	15	1/15 (6.7)	—	THE	After GC	25	IC-VIEW	N
Noma^48^	2017	R(PS)	Japan	136	6/68 (8.7)	15/68 (22)	MIE/Open	After GC	12.5	PDE	N
Ohi^49^	2017	R	Japan	120	1/59 (1.7)	9/61 (14.75)	MIE/Open	After GC	2.5	PDE	N
Pather^50^	2021	R	USA	100	6/100 (6)	—	MI-ILE	After GC	7.5	PINPOINT	T
Rino^28^	2018	R	Japan	33	5/33 (15)	—	3flnd	After GC	2.5	PDE	T
Sarkaria^51^	2014	P	USA	30	2/30 (6.7)	—	RAMIE	Before GC	10	Firefly	T/N
Schlottman^15^	2017	R	USA	5	0/5 (0)	—	Hybrid ILE	After GC	5	STORZ	T
Shimada^29^	2011	R	Japan	40	3/40 (7.5)	—	TTE	After GC	12.5	PDE	N
Slooter^52^	2020	P	NL	84	12/84 (14)	—	MI-ILE	After GC	0.05 mg/kg	Pinpoint/ spy	N/T
Talavera-Urquijo^4^	2020	P	Italy	100	32/100 (32)	—	MI-ILE	Before & After GC	0.3 mg/kg	Olympus	T
Thammineedi^34^	2020	P	India	13	0/13 (0)	—	MIE	After GC	2.5-15	PINPOINT	N
Von Kroge^3^	2020	R	Germany	20	7/20 (35)	—	Open	After GC	0.02 mg/kg	SPY Elite	T/N
Yamaguchi^53^	2021	P	Japan	129	4/129 (3)	—	Open/MIE	After GC	2.5	PDE	N
Yukaya^5^	2015	R	Japan	27	9/27 (33)	—	NS	After GC	0.1 mg/kg	HEMS	N
Zehetner^54^	2015	R	USA	144	24/144 (16.7)	—	MIE	After GC	2.5	SPY	N
LeBlanc^55^	2023	R	USA	312	4/61 (6,6)	13/251(5,2)	RAMIE	After GC	—	—	T
Shishido^56^	2022	R	Japan	39	7/39	—	MIE	After GC	10	PDE/ Firefly	N

AL, anastomotic leak; AS, anastomotic site; GC, gastric conduit; Inj, Injection; M K MIE, Mc Keown minimally invasive esophagectomy; MIE, minimally invasive esophagectomy; MI-ILE, minimally invasive Ivor lewis esophagectomy; MKE/ILE, Mc Keown esophagectomy/ Ivor Lewis Esophagectomy; N, neck; N, total numbers; NL, Netherland; NS, not specified; P, prospective; PDE- PhotoDynamic Eye; PS, Propensity Score Matching; R, retrospective; RA-ILE, Robotic-assisted Ivor Lewis Esophagectomy; SPY Elite System; T, thorax; LifeCell, Bridgewater, NJ, USA, IC-VIEW ^R^ Pulsion Medical System, Munich, Germany; Hamamatsu Photonics K.K, Firefly- Intuitive Surgical, Sunnyvale, CA, USA. PINPOINT- (Novadaq Technologies Inc. Richmond, Canada, 4CMOS laparoscopic fluorescence imaging system, Opman Mandi Company, Guangdong, China, Quest V2 Fluorescence imaging platform (Quest Medical Imaging), Middenmeer, The Netherlands, HyperEye Medical System (Mizuho Ikakogyo Co., Ltd, Tokyo, Japan, SPY Imaging System Novadaq, Ontario, Canada), laparoscopic camera (Olympus, Tokyo, Japan).

**Figure 4:**
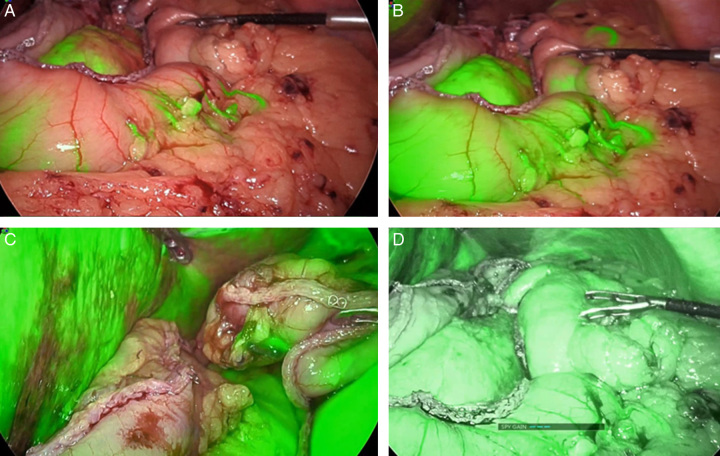
Indocyanine green (ICG) Fluorescence Angiography Assessment of Gastric Conduit Using Stryker’s 1688 AIM High-Definition Laparoscope. (A) Enhancement of the gastric conduit is observed after 7 sec of ICG injection in overlay mode. (B) Further enhancement is seen after 20 sec. (C) A close-up view reveals the avascular and dusky tip of the gastric conduit, along with the distal end of the divided oesophagus in overlay mode. (D) The gastric conduit is displayed in Spy env mode.

Aim of ICG-FA use in oesophageal surgery is to reflect the difference between subjective visual inspection and objective ICG-based assessment to identify the best site for EGA. Visual assessment of tissue perfusion during an esophagectomy may not consistently align with the adequacy of the blood supply. Data clearly indicate that ICG-FA results can differ from visual assessments. In few studies, ICG-FA revealed inadequate blood supply to the GC’s tip, despite visual assessments suggesting sufficient perfusion in 24–40% of the cases^15,43^. Figure 4A-D exemplarily shows ICG-FA assessment of the GC using Stryker’s 1688 AIM High-Definition telescope, demonstrating the avascular and dusky tip of the GC and oesophagus in overlay mode.

Changing the anastomotic site because of poor fluorescence at the original location, necessitating a revision of the GC and oesophageal stump is a challenging task but has been reported in some studies, with rates ranging from 6 to 40%^43,52,58^. A meta-analysis found that in the ICG-arm, the incidence of AL and graft necrosis (GN) was 11%, with a pooled change in management rate of 25%. This change in management included the resection of poorly perfused portions of the GC and a change in the anastomotic site. Subsequently, the ICG group exhibited a reduced incidence of AL and GN compared to the non-ICG group with a odds ratio of 0.30 (95% CI: 0.14–0.63)^57^.

Studies have reported the use of ICG in assessing reference point of well-perfused area of GC during esophagectomy for anastomosis. Campbell and colleagues utilized a reference point 10 cm from the pylorus and calculated FI 60 seconds after injecting the ICG dye. They considered areas with FI at least 75% of the reference point as well-vascularized and performed all anastomoses in these zones after necessary revision with consequently a reduced incidence of AL^37^. Kitagawa *et al.*^44^ observed a decrease in AL rate from 17.9 to 4.4% by visualizing the blood supply border of the RGEA using ICG-FA by the HyperEye Medical System and accordingly changing the anastomotic site.

Hence, we must assume that ICG-FA for assessing GC blood supply during esophagectomy has yielded promising results in reducing AL and GN incidence while aiding in optimal anastomotic site selection.

Assessment of the proximal oesophageal stump has also been addressed in few studies since patency of the EGA also equally depends on the oesophageal stump quality in determining AL^40,58^. Thammineedi *et al.*^58^ have demonstrated that ICG plays a definitive role in assessing the perfusion of the proximal oesophageal stump, necessitating revision in 40% of patients.

The use of ICG-FA has been proposed as a method to ensure good blood perfusion at the anastomosis site during surgery. Efforts have been undertaken to objectively stratify the ICG-FA findings which is essential for not only reporting but also replication and clinical implementation of the technique.

One such attempt represents the 90 sec to 60 sec rule. Kumagai *et al.*^46^ proposed a 90-second rule to ensure good blood perfusion at the anastomosis site during surgery. They created all anastomoses in the area of the GC that enhanced within 90-sec from the initial enhancement of the root of the RGEA. According to this rule, the tip needed resection in 50% of the cases, and the anastomotic site was changed in 52% (18 of 35 cases). None of the patients underwent anastomosis at a site with delayed enhancement after 90 seconds.

Yamaguchi and colleagues conducted a multicentric study and observed that the AL rate was 4.1% when EGA site occurred within 90 sec of enhancement and 2.4% when it happened within 60 sec using ICG-FA. They advocated the *“90-to-60-second rule”* with ICG-FA to prevent AL from EGA^53^. Pather and colleagues assessed the GC perfusion with ICG-FA under time constraints in minimally invasive Ivor Lewis esophagectomy. Segments of the GC that did not fully enhance within 60 sec were considered non-perfusion zones and were transected. Anastomosis was then performed between the distal native esophageus and the perfused proximal stomach during the thoracoscopic part of the procedure^50^.

Similar results have been published by Lou and colleagues in McKeown minimally invasive esophagectomy with AL of only 1.2% when anastomosis had been carried out in ICG-FA visualized zone within 60 sec. No vascular perfusion areas or perfusion times exceeding 60 sec indicated a poor tissue perfusion and presented higher AL rates of upto 10.4%^47^.

Since manual time measurement is subject to bias, hence attempts were made to evaluate the GC perfusion based on flow speed rate. ICG-FA was used by Koyanagi and colleagues to measure blood flow speed in the GC during surgery. They found a threshold of 1.76 cm/s for speed that predicted AL risk. Patients were divided into two groups based on the ICG flow speed—a simultaneous group and a delayed group. The simultaneous group had similar speeds in the GC wall and greater curvature vessels, while the delayed group had slower speed in the GC wall compared to the greater curvature vessels. None of the patients in the simultaneous group developed a AL, while 46.7% of the patients in the delayed group presented clinical evident ALs hence the group concluded that the ICG-FA using blood flow speed in GC is a useful tool to predict the risk of AL in oesophageal surgery^45^.

Interpretation of ICG-FA results remains a challenge as measurements can be subjective (evaluating FI and time to adequate fluorescence) or objective (assessing flow patterns, velocity, and inflow/outflow patterns via software analysis). Although desirable, objective measurements can be time-consuming due to calculations or software analysis. Van Den Hoven *et al*.^59^ identified 11 software programs for quantifying tissue perfusion via ICG near-infrared fluorescence imaging and stressed the importance of standardization for reliable results. The group categorized fluorescence studies into three types:

1. Static fluorescence analysis measures ICG fluorescence intensity in a specific region of interest to assess tissue perfusion but relies on camera settings and timing.
2. Dynamic fluorescence analysis with absolute intensity is valuable for objectively tracking changes in tissue perfusion over time.
3. Dynamic normalized fluorescence expresses fluorescence intensity as a percentage change from maximum intensity over time.

Ishikawa and colleagues performed a post hoc analysis and compared the FI curves at the antrum to the FI curves at the tip of the GC and an area 5 cm distal/ caudad to the GC tip. They found a significantly lower max FI at the tip and 5 cm distal/ caudad to the tip were associated with AL, and time to max FI at 5 cm distal to the tip was associated with AL^42^.

Time-fluorescence intensity curves graphically display the intensity of fluorescence over time and are an effective tool for evaluating tissue perfusion and detecting abnormal pattern, particularly delayed ICG fluorescence flow, which can indicate inflow or outflow issues. Recent research has shown that blood flow decreases significantly over time from the GC creation phase to the anastomotic phase, and tension or compression from pulling up the GC through the posterior mediastinal or retrosternal route may also affect blood perfusion. Time-fluorescence intensity curves can thus offer valuable insights into tissue perfusion and help surgeons detect potential issues during procedures. Recently Galema and colleagues demonstrated three pattern using ICG-FA for GC evaluation - 1 (steep inflow, steep outflow); 2 (steep inflow, minor outflow); and 3 (slow inflow, no outflow). Major finding of that study was to outline the poor inter-observer agreement^41^. Yukaya *et al.*^5^ also differentiated three curves (normal, inflow delayed, outflow delayed) and their association with AL rates. Although no significant correlation could be established between AL and blood flow pattern in that study with only 27 patients, they were able to produce a reproducible quantitative measurement tool. The study by Ishige *et al.*^60^ reported no AL in 20 patients but outlined the importance of multiple quantitative measurements as they could clearly show a decrease between quantitative FI between the time of GC creation and anastomosis while macroscopically no obvious difference was notable.

In recent years robotic-assisted minimally invasive esophagectomy (RAMIE) has gained much popularity hence it is necessary to address the role of ICG-FA in RAMIE as well although the current available data with focus on this topic is very limited. De Groot *et al.*^39^ reported in a prospective study change of anastomotic site in 14% (9 out of 63). Sakaria and colleagues assessed ICG-FA during RAMIE and reliably identified the vascular arcade termination in all 30 study patients. Furthermore, they reported on even previously unseen small transverse vessels becoming visible with ICG-FA in RAMIE. ICG-FA also aided in determining the vascular arcade’s position during greater curve and omentum mobilization, enhancing surgical precision and safety^51^. Hodari *et al.*^40^ investigated the effectiveness of Firefly and ICG-FA in identifying perfusion demarcation during RAMIE. Integrated Firefly accurately pinpointed perfusion zones in all included 54 patients, especially near the oesophageal stump. The use of ICG-FA reduced AL rates from 20% to 0%, underscoring its value in improving patient outcomes utilizing robotic platform. In line with this is a most recent report by LeBlanc and colleagues outlining the impact of ICG-FA in RAMIE as it directly impacts surgeon’s decision-making (80%!) for additional resection of the GC. In addition, they demonstrated a correlation between elevated time to initial perfusion and maximum perfusion with ALs^55^.

### ICG-FA and reduction in anastomotic leaks

ICG-FA has become an increasingly valuable tool for assessing tissue perfusion during esophagectomy. Its use in evaluating GC perfusion before anastomosis has been shown to significantly reduce the risk of AL. Efforts have been made to position the anastomotic site in a well-perfused area to decrease AL rates. ICG-FA has been shown to be an independent risk factor for AL^49^. Placing the anastomosis in an area of good perfusion, also referred to as the “optizone,” has been proven to decrease the rate of AL. When the anastomotic site was positioned within the optizone as defined by ICG-FA several authors reported reduction of AL rates in upto 45%^37,50,54^. In addition, Noma *et al.*^48^ not only noted a significant reduction in the incidence but also severity of AL with the use of ICG-FA.

Two meta-analyses found a 70% overall risk reduction for AL with the use of ICG-FA^57,61^. In addition, a meta-analysis by Slooter reported even 70% risk reduction in GC necrosis with ICG-FA^57^. These findings are also supported by a recent a systematic review by Van Dele *et al.*^62^. In addition a propensity score matched study by Shishido *et al.*^56^ clearly outlined the benefit of ICG-FA with AL rate of 9% in the ICG-FA group.

On the contrary we must also acknowledge reports questioning the efficacy of ICG-FA in terms of reducing ALs. A meta-analysis by Casas *et al.*^63^ suggested that using ICG-FA does not appear to reduce AL rates in patients undergoing minimally invasive esophagectomy with intrathoracic anastomosis. Another meta-analysis by Zhang and colleagues reached to a similar conclusion. ICG-FA was declared by the group only to be useful for reduction in ALs and consequently shorter postoperative hospital stay for patients undergoing cervical anastomosis only. However, it was not found to be effective for patients undergoing intrathoracic anastomosis as reported by Cases and colleagues too. Additionally, the application of ICG fluorescence both before and after GC creation was more effective in preventing AL^64^.

Despite the adoption of ICG-FA, the risk of AL persists as other factors contributing to ALs remain as potential morbidity factors. Various meta-analyses have reported AL incidence rates ranging from 11% to 14% following intraoperative ICG-FA^52,61^. Management strategies regarding post-ICG assessment have involved resecting poorly perfused GC and changing the anastomotic site. However, despite these interventions, the rates of ALs and GN remain elevated in general. In fact, one meta-analysis found that the pooled incidence of AL and GN increased after management changes, potentially due to anastomotic tension and selection bias among patients with poor vascularization or vasculature status^57^.

It is important to note that most studies evaluating EGA with ICG assessment are retrospective and involve small sample sizes. Very few have included a control group without ICG assessment^37,38,43^. To provide a more robust evaluation of ICG-FA effectiveness in reducing AL incidence, larger prospective randomized controlled trials are needed.

In addition, AL following esophagectomy depends not only on adequate perfusion but also on factors like anastomotic tension, location, and surgical approach and patient overall condition. Numerous studies have uncovered various risk factors associated with ALs. Kassis *et al.*^9^ linked ALs to obesity and comorbidities like congestive heart failure, hypertension, and renal insufficiency, as well as the type of anastomosis performed. Similarly, Hall *et al.*^65^ identified increased operative time, elevated preoperative white blood cell count, pre-existing diabetes, and perioperative transfusion as independent AL risk factors. With regard to the GC Pather *et al.*^50^ highlighted the non-perfusion areas as independent AL risk factors. However, some authors, as Yamaguchi *et al.*^53^ did not find any significant differences in AL rates and reconstruction route, anastomotic method, tumour location, or the administration of preoperative chemotherapy or radiation therapy.

Table 2 summarizes various studies on ICG-FA conducted to date and their outcomes, providing a comprehensive overview on the research in this field.

The complexity of anastomosis healing does not allow to cover all aspects in a single review article hence we need to address the limitations that need to be acknowledged. Firstly, it is essential to note that this is not a meta-analysis or a systematic review; rather, it offers a summary of the existing literature on the subject. Systematic reviews and meta-analyses are undoubtedly a tool to overcome sample size, heterogeneity, and outcome reporting. Both allow to evaluate an information available for decision-making. On the other hand, the quality of systematic reviews and meta-analyses depend to greatest extent upon the addressed question with inevitable selection bias and loss of information. The systematic reviews and meta-analyses we have included in this paper for example report on outcomes based upon 9–25 studies. Furthermore, many of the articles included in those systematic reviews and meta-analyses were characterized by very small sample sizes, retrospective designs, and a potential for publication bias (type of reconstruction!). Additionally, the absence of control groups in most studies limits the ability to draw definitive conclusions. Moreover, variations in ICG dosages, techniques, and imaging systems used among the studies add another layer of complexity to the interpretation of the findings. Finally, it is important to highlight that high-quality RCTs on this subject are lacking. These limitations emphasize the need for further research to establish more robust evidence regarding the effectiveness of ICG-FA in the context of GC and EGA perfusion assessment. At present majority of the data available pinpoints towards a benefit of ICG-FA in oesophageal resection with GC reconstruction. Although many qualitative and quantitative tests have been evaluated to objectively stratify ICG-FA results in GC and EGA perfusion assessment, none can be designated as the gold-standard since each one has only been tested in a single study and comparative analysis are missing till date. However, this is ultimately to be happen, especially within the frame of robotic platforms with included ICG-FA tools becoming more and more popular.

We aimed here to cover the entire topic related to ICG-FA—from applied dosage, imaging systems, assessment and stratification tools, and outcome—in GC and EGA perfusion assessment.

## Conclusion

In summary, ICG-FA stands as a safe and valuable tool for assessing GC and EGA perfusion during esophagectomy. It furnishes crucial information to guide GC construction and anastomotic site selection, potentially reducing the occurrence of AL. However, it’s essential to recognize that AL development is influenced by various factors. Therefore, ICG-FA should be employed in conjunction with other strategies to optimize both patient-specific and procedural factors, ultimately minimizing AL rates. Altogether, the utilization of ICG-FA represents a promising advancement in enhancing the safety and effectiveness of oesophageal surgery.

## Ethical approval

None.

## Consent

None.

## Source of funding

None.

## Author contribution

S.N.: design - literature search, data extraction, writing—final proof. P.K.: - literature search, data extraction, writing—final proof. S.S.: design—data extraction, writing—final proof. A.D.: literature search, data extraction, final proof. M.B.: literature search, data extraction—final proof. P.J.: literature search, data extraction, final proof. M.A.: revision literature research, data extraction, final proof. S.A.: literature search, data extraction, final proof. T.A.M.: data extraction, final proof. D.C.B.: revision, final proof. K.V.V.N.R.: writing, final proof. T.S.R.: design, writing, final proof. Y.K.V.: design, literature search, data extraction, writing, final proof.

## Conflicts of interest disclosure

Not invited.

## Research registration unique identifying number (UIN)

Not invited.

## Guarantor

Syed Nusrath. Yogesh Vashist.

## Data availability statement

Not invited.

## Provenance and peer review

Not invited.

## Data statement

This is a review based on available data in the literature. We have summarized the results of the studies published in this paper. We can provide that data upon requirement. Also, data from our own institute can be provided to the extent of this review. Due to copyright issues, we cannot provide the full-length articles, but the reference list of the paper, represents the literature from which data is included.
